# Cleaner fish with mirror self-recognition capacity precisely realize their body size based on their mental image

**DOI:** 10.1038/s41598-024-70138-7

**Published:** 2024-09-11

**Authors:** Taiga Kobayashi, Masanori Kohda, Satoshi Awata, Redouan Bshary, Shumpei Sogawa

**Affiliations:** 1 Laboratory of Animal Sociology, Department of Biology, Graduate School of Science, Osaka Metropolitan University, Osaka, Japan; 2 Laboratory of Animal Sociology, Department of Biology and Geosciences, Graduate School of Science, Osaka City University, Osaka, Japan; 3 Institute of Zoology, University of Neuchâtel, Neuchâtel, Switzerland

**Keywords:** Private self-awareness, Mental image, Mirror self-recognition, Body size awareness, Mirror use, *Labroides dimidiatus*, Behavioural ecology, Animal behaviour, Psychology

## Abstract

Animals exhibiting mirror self-recognition (MSR) are considered self-aware; however, studies on their level of self-awareness remain inconclusive. Recent research has indicated the potential for cleaner fish (*Labroides dimidiatus*) to possess a sophisticated level of private self-awareness. However, as this study revealed only an aspect of private self-awareness, further investigation into other elements is essential to substantiate this hypothesis. Here, we show that cleaner fish, having attained MSR, construct a mental image of their bodies by investigating their ability to recall body size. A size-based hierarchy governs the outcomes of their confrontations. The mirror-naïve fish behaved aggressively when presented with photographs of two unfamiliar conspecifics that were 10% larger and 10% smaller than their body sizes. After passing the MSR test, they refrained from aggression toward the larger photographs but still behaved aggressively toward the smaller ones without re-examining their mirror images. These findings suggest that cleaner fish accurately recognize their body size based on mental images of their bodies formed through MSR. Additionally, mirror-experienced fish frequently revisited the mirror when presented with an intimidating larger photograph, implying the potential use of mirrors for assessing body size. Our study established cleaner fish as the first non-human animal to be demonstrated to possess private self-awareness.

## Introduction

Self-awareness refers to the capacity to focus on oneself and identify one’s own existence^1–4^. The mirror self-recognition (MSR) test, which assesses the ability to recognize one’s mirror reflection as oneself, is regarded as the most reliable evidence of self-awareness^5–7^. To attain MSR, individuals must have a degree of comprehension of their own existence^1,8–10^. MSR ability is demonstrated through the mark test, wherein subjects are surreptitiously provided with an exotic mark on their directly invisible body parts and subsequently observed for detection and attempts to touch or remove the mark only when exposed to a mirror^5,6,11^. Several vertebrates have passed the test and have been demonstrated to possess MSR ability, including the great apes^5,12,13^, dolphins^14–16^, elephants^17,18^, Eurasian magpies^19,20^, and cleaner fish^21,22^. In other words, these animals are considered to possess self-awareness^4,7,8,23,24^.

However, the level of self-awareness in animals that possess MSR ability remains controversial due to the inconclusive evidence provided by the mark test regarding private self-awareness^2,7,25–28^. Self-awareness is categorized into two classes based on the nature of self-information being processed^3,7,29,30^. The fundamental level is called public self-awareness, which enables individuals to focus on perceptual self-aspects observable by others, such as behavior or appearance^3,29,30^. Private self-awareness, which represents a more sophisticated level, allows individuals to focus on their internal aspects; that is, mental states, such as mental images, goals, self-memories, perceptions, intentions, and standards^3,29,30^. Some researchers regard MSR as an indicator of public self-awareness and object to the inclusion of private self-awareness in its interpretation^7,27,31,32^. For the first exposure to a mirror, individuals attain MSR by realizing the behavioral contingency between themselves and their mirror reflection, a hypothesis called the kinesthetic visual matching mechanism^31,32^. In this case, they focus on their own behavior, and this process can be interpreted through public self-awareness^7,27,31,32^. Conversely, once individuals have established MSR, they can recognize their mirror images as themselves without re-examining the contingency, like humans^2,9,25^. In this case, they maintain their visual image in their minds, suggesting evidence of private self-awareness^2,3,7,25,26^.

Recently, Kohda et al. showed that cleaner fish construct a mental image of their face by attaining MSR^33^. The authors excluded the interpretations involving the kinesthetic visual matching mechanism through compelling experiments using static photographs^33^. However, cleaner fish may possess a mental image specialized only for the face as a byproduct of their sophisticated individual recognition ability; they also construct facial representations of other conspecifics^33^. Furthermore, they possess the ability to distinguish numerous individuals of other species that interact in the wild^34^. Therefore, additional evidence regarding mental states is required to substantiate the idea that cleaner fish possess a general form of private self-awareness. In the present study, we focused on the ability to perceive body size, which is crucial for cleaner fish to survive in a competitive social environment^35,36^.

Body size significantly influences the behaviors of cleaner fish, particularly in intraspecific competition^35,36^. They establish a dominance hierarchy depending on body size within a stable group^36,37^. In general, larger individuals tend to have an advantage in confrontations, whereas smaller ones often incur costs such as injuries^38^. Before engaging in physical aggression against an unknown conspecific rival, cleaner fish often swim in parallel, presenting one side of their bodies to their opponent^35^. Through parallel swimming, fish may assess the approximate body size of their opponents as an indicator of their competitive ability, which may reduce the likelihood of futile conflicts^38–40^. In this context, fish can only estimate their approximate sizes relative to those of their opponents. However, after cleaner fish recognize their mirror reflection as themselves, they are able to estimate their body size more accurately by swimming parallel to the mirror, thereby enhancing precision in comparing their body size to that of a rival. Therefore, we investigated whether cleaner fish possessed the ability to recognize body size recalled from a mental image of their body.

**Figure 1:**
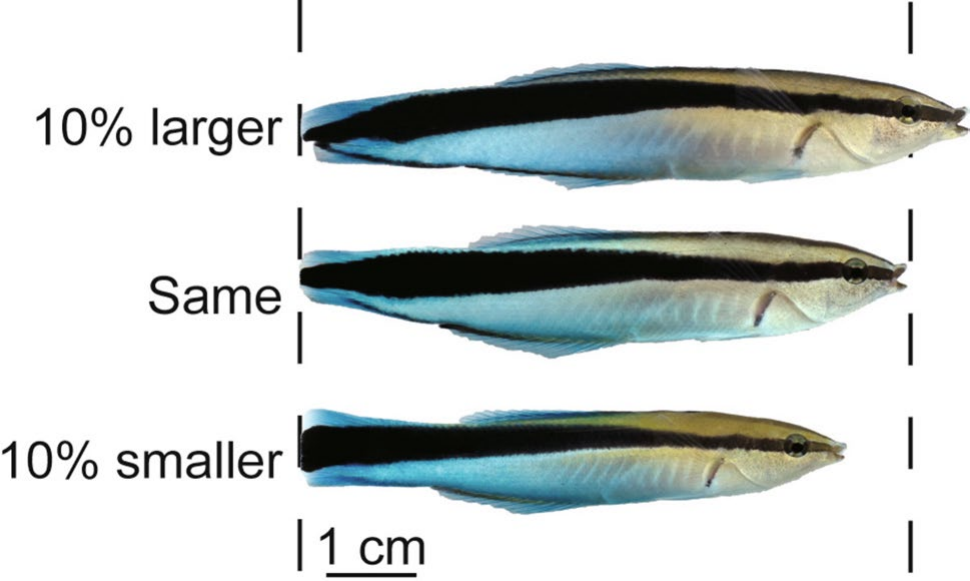
An example of a series of photographs presented to the focal fish. The top photograph was enlarged by 10% relative to the actual size of the focal fish, the middle photograph was resized accordingly, and the bottom photograph was reduced to 10% of its actual size. These photographs were aligned to orient the heads to the right and adjusted for brightness, contrast, and exposure as necessary. None of the focal fish encountered any individuals in these photographs.

**Figure 2:**
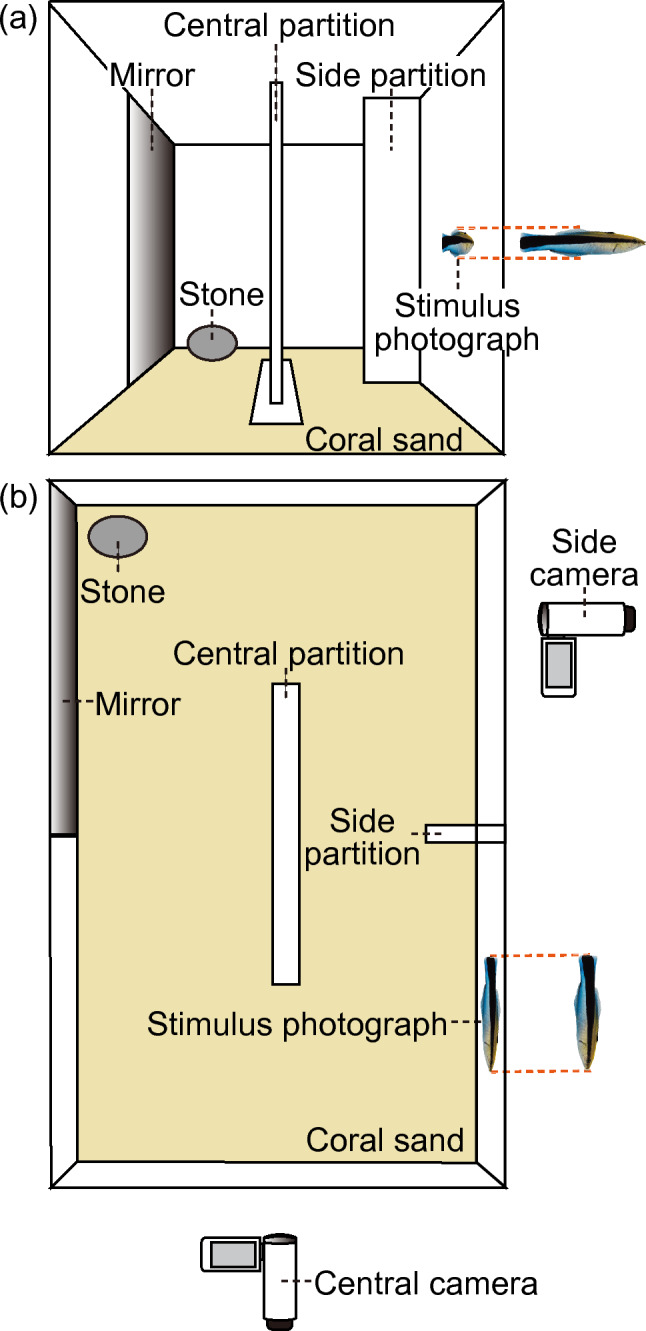
Schematic diagram of an experimental aquarium (450 × 300 × 300 mm^3^): (a) frontal perspective and (b) aerial perspective. A photograph was displayed on the anterior right side of the aquarium wall from the outside. A mirror (225 × 260 mm^2^) was positioned on the posterior left section of the aquarium wall from the exterior. An opaque partition (150 × 260 mm^2^) was centrally placed within the aquarium to prevent the focal fish from simultaneously observing both the photograph and the mirror. An additional opaque side partition (50 × 260 mm^2^) was attached behind the photograph to enable the fish refuge from the photograph. A small stone was positioned in front of the mirror to allow the fish to scrape their throats in the mark test. The behaviors of the fish were recorded using two cameras: a central camera to capture the entire aquarium and a side camera to monitor the blind spot behind the side partition.

We conducted a series of laboratory experiments to test this hypothesis. We prepared two sets of photographs of different cleaner fish that were 10% larger, equal in size, and 10% smaller than the focal fish (Fig. 1). None of the focal fish had previously encountered any individuals in these photographs. In the first session, we presented one set of photographs to 15 fish. Eight fish in the mirror treatment group were exposed to a mirror for one week following the first photograph presentation. The seven others in the control group were maintained for an equivalent duration without mirrors. In the second session, we presented another set of photographs. We predicted that the focal fish, having attained MSR, would construct an internal representation of their body size and refrain from aggressive interactions with larger photographs in the second session. In contrast, we expected that mirror-naïve fish would lack accurate awareness of their own body size and exhibit aggression toward all photographs in both the first and second sessions. Crucially, the focal fish were prevented from simultaneously observing both the photograph and the mirror by the partition placed in the experimental aquarium (Fig. 2). Therefore, they would need to visit the mirror and the photograph alternately to compare their body size with that of the opponent. We also examined their behavior toward the mirror to determine the likelihood of their intentional mirror use. We aimed to identify the constituent elements of mental states other than the mental image of the self-face in order to establish that cleaner fish possess private self-awareness.

## Results

### The behavior of the focal fish toward the photographs

When presented with a photograph, the focal fish closely approached and often gazed at it for durations ranging from 0.5 to 2.0 s, with their dorsal and caudal fins spreading. This behavior is similar to the frontal display that fish often exhibit against competitors^35^. We defined this behavior as aggressive behavior against the photograph (Supplementary Movie 1; refer to Behavioral analysis in the Materials and methods). The longer the focal fish engaged in this behavior, the less likely they were to perceive the photograph as a formidable threat. We measured the duration of the initial aggressive interaction of the focal fish toward the photographs, as previous research has demonstrated that fish rapidly discriminate between photographs^41^. We compared this duration of aggressive behaviors among treatments (mirror and control), sessions (first and second presentation with the photographs), and photograph sizes (larger, the same, and smaller).

**Figure 3:**
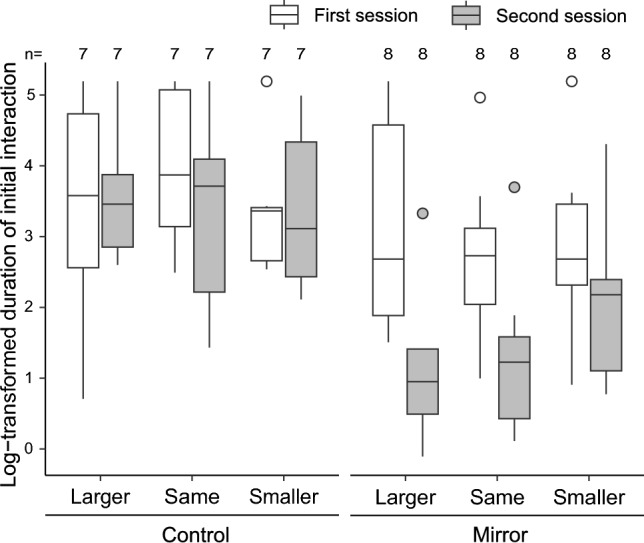
The initial duration of aggressive interaction exhibited by the focal fish toward the photographs. The horizontal axis depicts the size of the photographs, whereas the vertical axis represents the log-transformed duration of the initial aggressive behavior exhibited by the focal fish. The black lines within each box plot display the medians, with the upper and lower boundaries indicating the 75th and 25th quartiles, respectively. Whiskers above and below the box represent the maximum and minimum observed values, respectively. Dots denote outliers. The two white dots on the right boxes labeled 'Same' and 'Smaller,' as well as the two dark dots on the right boxes labeled 'Larger' and 'Same,' were each derived from the same individual. The six boxes on the left and right represent outcomes from the control and mirror treatment groups, respectively. White boxes indicate the results of the first session, whereas the dark ones indicate those of the second session. Numbers above the box plot denote the sample size.

Consequently, we found a significant interaction between treatment and session [Fig. 3, linear mixed model (LMM), F_1,65_ = 9.45, *p* = 0.003, partial *η*^*2*^ = 0.13 (95% CI: 0.03, 1.00)]. Prior to encountering the mirror, the focal fish in the mirror treatment group exhibited aggressive behavior toward the photographs, regardless of size [see the three right white boxes in Fig. 3, LMM: F_2,14_ = 0.56, *p* = 0.58, partial *η*^*2*^ = 0.07 (95% CI: 0.00, 1.00)]. Having adequately interacted with a mirror, all mirror-experienced fish scraped their marked throat at least once and successfully passed the mark test (2.5 times ± 0.63 SEM/ 2 h, n = 8). After attaining MSR, they spent significantly less time engaging in aggressive interactions with photographs than when they were mirror-naïve [emmeans, t ratio = 5.16, df = 65, *p* < 0.0001, partial *η*^*2*^ = 0.29 (95% CI: 0.15, 1.00)]. When specifically analyzing fish that established MSR, the relative photograph size had a significant effect on the initial aggressive interactions [see the right three dark boxes in Fig. 3, LMM, F_2,14_ = 13.60, *p* = 0.0005, partial *η*^*2*^ = 0.66 (95% CI: 0.34, 1.00)]. They behaved less aggressively with the larger [emmeans, t ratio =  − 4.96, df = 14, *p* = 0.0006, partial *η*^*2*^ = 0.64 (95% CI: 0.33, 1.00)] and the same-sized photographs [emmeans, t ratio =  − 3.88, df = 14, p = 0.004, partial *η*^*2*^ = 0.52 (95% CI: 0.19, 1.00)] compared to the smaller one. In contrast to the mirror-experienced individuals, the focal fish in the control group remained aggressive throughout the experiment [emmeans, t ratio = 0.62, df = 65, *p* = 0.54, partial *η*^*2*^ = 0.006 (95% CI: 0.00, 1.00)].

### The behavior of the focal fish to the mirror

**Figure 4:**
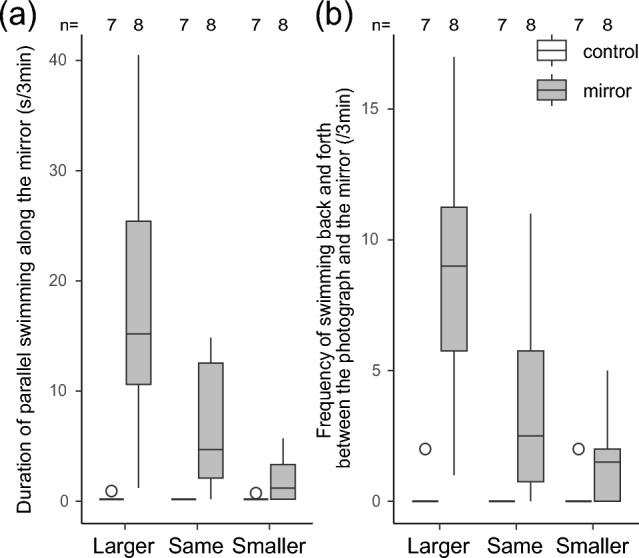
The mirror inspection behaviors exhibited by the focal fish: (a) the cumulative duration of parallel swimming along the mirror over 3 min and (b) the total number of times the fish swam back and forth between the photograph and the mirror within 3 min. The horizontal axis represents the size of the photographs. Black lines within each box represent the median values, with the upper and lower boundaries of the box denoting the 75th and 25th quartiles, respectively. Whiskers extending above and below the box indicate the maximum and minimum observed values. Dots represent outliers. White boxes depict outcomes from the control treatment group, while dark ones illustrate those from the mirror treatment group. The number above the box plot denotes the sample size.

After mirror exposure, the focal fish left the site of the photograph, visited the mirror, and repeatedly swam parallel to the mirror surface (see Supplementary Movie 2, the dark boxes in Fig. 4a). In contrast, the fish in the control group almost never exhibited those behaviors (0.062 s ± 0.043 SEM, n = 7, see the white boxes in Fig. 4a). The relative photograph size significantly affected the duration of parallel swimming [LMM, F_2,14_ = 12.20, *p* = 0.0009, partial *η*^*2*^ = 0.64 (95% CI: 0.30, 1.00)]. The fish that established MSR swam in parallel for a longer duration when presented with a larger photograph compared to either the same size one [emmeans, t ratio = 2.86, df = 14, *p* = 0.032, partial *η*^*2*^ = 0.37 (95% CI: 0.06, 1.00)] or the smaller one [emmeans, t ratio = 4.92, df = 14, *p* = 0.0006, partial *η*^*2*^ = 0.63 (95% CI: 0.33, 1.00)].

In addition, the focal fish that established MSR often swam straight to the mirror, paused momentarily, or swam parallel to the surface, and then immediately returned directly to the site of the photograph (six out of eight for smaller photographs, six out of eight for same-sized ones, and eight out of eight for larger ones; see Supplementary Movie 3, the dark boxes in Fig. 4b). In contrast, fish in the control group rarely exhibited frequent reciprocation (see the white boxes in Fig. 4b). The frequency of swimming back and forth between the photograph sites and the mirror was influenced by the relative photograph size [LMM, F_2,14_ = 24.76, *p* < 0.0001, partial *η*^*2*^ = 0.78 (95% CI: 0.55, 1.00)]. The fish that established MSR swam back and forth more frequently in the presence of a larger photograph compared to either the same size one [emmeans, t ratio = 4.53, df = 14, *p* = 0.001, partial *η*^*2*^ = 0.59 (95% CI: 0.28, 1.00)] or the smaller one [emmeans, t ratio = 6.93, df = 14, *p* < 0.0001, partial *η*^*2*^ = 0.77 (95% CI: 0.55, 1.00)].

## Discussion

Our study shows that the focal fish behaved aggressively toward all three sizes of photographs without differences before their mirror experience but exhibited less aggression toward the larger and same-sized photographs compared to the smaller ones after attaining MSR. Together with the control treatment, the findings indicate that mirror-experienced fish may reduce their aggression not merely due to habituation to the photographs presented twice but rather by improving their ability to discriminate 10% size differences between themselves and the photograph by attaining MSR. Notably, there is a possibility that the focal fish immediately determined whether to approach or leave the photograph without revisiting the mirror, whereas the central partition prevented them from simultaneously comparing their mirror reflections with the photograph (Fig. 2). These findings suggest that cleaner fish construct a mental image of their body based on their mirror reflection by attaining MSR and make a decision whether to attack the photograph based on this mental representation. This implies that the focal fish were likely to use their internal images as a standard to assess the size hierarchy between themselves and the photographs. These findings suggest that cleaner fish would likely possess two mental states: a mental image of their body and internal standards for comparing body size^3^.

After leaving the photograph site, some focal fish that established MSR repeatedly swam parallel to the mirror. This parallel swimming is analogous to the behavior of cleaner fish observing their mirror-reflected bodies in previous studies^21,22^. This behavior is also similar to the lateral display, in which cleaner fish compare their body size relative to that of their opponent^35,39,40^. A previous study demonstrated that fish could assess their relative body size differences from their competitors without direct physical interactions^42^. Given that the focal fish had only visual cues, such as body size, it is possible that they engaged in an extensive examination of their own body size using their mirror image as a point of reference. Thus, it appears that cleaner fish visually recognize their body size by swimming in parallel with their mirror images.

When a larger photograph was presented, the cleaner fish that established MSR swam parallel along the mirror longer and swam back and forth between the sites of the photograph and the mirror more frequently. These findings suggest that these fish may have assessed their body size to decide whether to exhibit aggressive behavior toward the intimidating larger photograph. If they were merely attempting to scrutinize their body size in the photographs, they would have increased these behaviors when presented with a photograph of equal size, which represents the smallest size difference in comparison to themselves. It is unlikely that they merely fled from the photograph since they repeatedly returned to the larger photograph. Once they avoid larger and same-sized photographs, they appear to persist in interacting with their opponent and may elaboratively compare their body size relative to that of the photograph. Using their mirror image to assess their body size would require considerable time, particularly when confronted with a more formidable, larger photograph than that of the same size. These findings imply that cleaner fish establish MSR use their mirror images with the intention of reassessing their body size and seem to observe the mirror not for the purpose of scrutinizing their body size but rather to decide whether to initiate aggression. These interpretations suggest that cleaner fish may possess two mental states: intentions and goals^3^.

MSR is the most influential paradigm for demonstrating self-awareness in animals^24,26,43^. However, the conventional mark test, the most widely used method for investigating MSR, is unreliable for individuals or species demonstrating little interest in an ecologically meaningless mark and lacking motivation for its removal^11,22,33,44^. Nevertheless, failure is often interpreted as evidence of deficient self-awareness. To steadily evaluate the self-awareness of animals, a more widely applicable method that can elicit the motivation of animals to solve the task using their ecological characteristics is required^10,11^. Our study used the size principle in fish: individuals of different body sizes establish a size-dependent dominance hierarchy, whereas individuals of similar body sizes establish exclusive territories. This phenomenon is often observed in fish inhabiting stable social hierarchies, including cleaner fish^36^. Assessing relative body size may be easy for cleaner fish when the relative difference in body size is obvious (e.g., a 20% difference in size; refer to Supplementary Results), but this may be challenging with subtle differences. The focal fish that established MSR refrained from displaying aggressive behavior toward an opponent which they exhibited high aggression when mirror-naïve. If similar behavioral patterns are detected in other fish species under analogous experimental conditions, it may be inferred that these fish recognize their mirror images as themselves without conducting the mark test. In this manner, the present study proposes an alternative approach to demonstrate MSR and, thereby, self-awareness in a wide range of fish species with a dominance hierarchy based on body size.

This method can be further applied to conventional experiments on body size awareness. Animals with body size awareness possess knowledge of their relative body size as a representation formed visually or proprioceptively through daily experiences. Consequently, they can easily select an appropriate passageway without prior practice^45,46^. This method has the advantage of being simple enough to be applicable to a wide range of species; for example, human infants^45^, dogs^47,48^, crows^49^, snakes^50^, and even bumblebees^51,52^. However, the conventional procedure for demonstrating body size awareness does not reveal self-directed behavior^11,26,43^ and is difficult to use as convincing evidence of self-awareness. Self-directed behavior refers to instances in which animals investigate or manipulate personal features, such as self-exploratory behavior using a mirror^1,11,26^. Indeed, self-directed behavior (e.g., re-examining one’s body size after failing to pass through a hole) has not been documented in the abovementioned studies. When the hole size closely approximates that of the subjects, they will encounter challenges in determining whether to pass through it. If they can correctly determine whether to pass through the gap closer in size to themselves after sufficient exposure to a mirror, or if they reassess their reflection when confronted with such a challenging task, it could be interpreted as self-directed behavior and suggestive of their possession of self-awareness. This procedure will be applicable to animals that use visual perception to assess body size.

The present study suggests that cleaner fish construct a mental image of their body after attaining MSR and make decisions regarding confrontations based on this mental representation. Cleaner fish would likely use mirrors to confirm their body size. This study makes a noteworthy contribution by accumulating evidence of four mental states, in addition to the mental image of faces, thereby providing more robust support for the hypothesis that cleaner fish possess private self-awareness. Cleaner fish will be the first non-human animals to be shown to possess private self-awareness. We propose a versatile method to test self-awareness, particularly extending to private self-awareness, without the need for the mirror mark test. If self-awareness is revealed in more animals than is currently acknowledged, we will be able to examine a more reliable evolutionary hypothesis on self-awareness. Interpreting the differences in the focal fish's responses to the photographs and the mirror through operant conditioning is challenging because they did not appear to receive any reinforcement for their responses. Instead, they may adjust their level of mirror image inspection based on uncertainty concerning their body size. These implications suggest that cleaner fish may possess metacognition, which refers to the awareness of their cognitive states^4,24,53^. Metacognition is considered a component of self-awareness equivalent to self-recognition^4,24^. Further research is required to ascertain whether cleaner fish possess this highest level of cognitive capacity. Our research substantiates the likelihood that cleaner fish possess private self-awareness and provides clues regarding the potential for metacognition. The present study will resolve disputes about the level of self-awareness in animals and serve as a pivotal milestone for future research to elucidate the evolutionary pathway of self-awareness.

## Materials and methods

### Subject animals and housing

Cleaner wrasse (*Labroides dimidiatus*) is a monandric protogynous teleost fish that lives in coral reef habitats with complex intra- and inter-specific social structures^36,54^. Males establish their harems, which include several females, and a strict size-dependent dominance hierarchy maintains social relationships among individuals^36,37^. In particular, females of similar body sizes have exclusive territories, whereas those of different body sizes occasionally overlap in their territorial boundaries^36,54^. Cleaner fish primarily feed on ectoparasites or mucus on the surface of client fish that visit their territories^55,56^. Therefore, defending their territory against conspecific rivals of similar size is essential for their survival. They have approximately 2,300 daily interactions with more than 100 species^55,57^ and can discriminate between individual clients^34^. Such complex social interactions are considered to develop various sophisticated cognitive capacities in addition to MSR^21,22,33^, such as tactical deception^58,59^ and transitive inference^60,61^. They also provide rudimentary evidence of episodic-like memory^62^ and theory of mind^63^.

We used 15 wild cleaner fish, ranging in total length from 58 to 83 mm (mean ± SD: 67.7 mm ± 6.8), obtained from a commercial supplier. As they were smaller than the minimum size of males, we estimated them as female^21,36^. Eight individuals (68.9 mm ± 7.6 SD) were used for the mirror treatment, during which they were exposed to a mirror between two sessions of photograph presentation. The remaining seven individuals (66.4 mm ± 6.0 SD) were used for the control treatment and were not exposed to a mirror throughout the experiment. The average total length of the focal fish did not differ between treatments (unpaired Student t-test, t = 0.68, df = 13, *p* = 0.51). They were individually housed in their experimental aquaria (450 × 300 × 300 mm^3^; Fig. 2) and maintained under a 14-h light: 10-h dark cycle in the laboratory of Osaka City University (Osaka Metropolitan University). A PVC tube (150 mm in length) was used as a sleeping shelter. The bottom of the aquarium was covered with fine coral sand. The water temperature was maintained at 24–26 °C, and the water was aerated and filtered to maintain optimal conditions. The fish were daily supplied with small portions of diced fresh prawn in the morning and artificial flake food (TetraMin®) in the evening. The conditions under which the cleaner fish were kept were identical to those used in previous studies conducted in our laboratory^21,22,33^.

### Stimulus photographs

We took photographs of 12 cleaner fish that had not been encountered by any focal fish using a Nikon D610 digital camera and edited them using the image editing software GIMP ver. 2.10.18 (https://download.gimp.org/gimp/v2.10/windows/). Eight distinct photographs were randomly selected from the total set of 12 photographs for each focal fish. We cut photographs along the outline, aligned them to orient their heads to the right, and adjusted for brightness, contrast, and exposure as necessary. Subsequently, we resized the photographs into three distinct patterns based on the total length of each focal fish: two larger photographs were enlarged by 10%, two smaller photographs were reduced by 10%, and four photographs were of equal size (Fig. 1). A 10% size difference would be an appropriate threshold as mirror-naïve fish did not exhibit aggression toward conspecific photographs that were 20% larger than themselves but still engaged in aggression toward photographs that were 10% larger in preliminary trials (Supplementary Results). The photographs were printed on commercially available photographic paper using an inkjet printer (EPSON EP-30VA), cut along the outline, and laminated.

### Experimental aquaria

Figure 2 shows the experimental aquarium (a: frontal perspective, b: aerial perspective). The photograph was attached to a white polypropylene plastic sheet (225 × 260 mm^2^) and presented at the center of the anterior right side of the aquarium wall from the outside. A mirror (225 × 260 mm^2^) was positioned on the posterior left section of the aquarium wall from the exterior. An opaque plastic sheet (225 × 260 mm^2^) was interposed between the mirror and the aquarium wall to shield the focal fish from observing the mirror surface until the end of the first session of photograph presentation in the mirror treatment and throughout the control treatment. The central (150 × 260 mm^2^) and side partitions (50 × 260 mm^2^) were constructed of opaque white corrugated plastic boards. The central partition was placed at the center of the aquarium as a visual barrier between the mirror and the photograph, whereas the side partition was attached behind the photograph to enable the focal fish to take refuge from the photograph. A small stone was placed in front of the mirror, which enabled fish in the mirror treatment group to scrape their throats during the mark test. This behavior provides evidence that cleaner fish are sufficiently exposed to the mirror to attain MSR^21,22^. We recorded the behavior of all focal fish in the presence of a photograph and mirror-experienced fish during the mark test using two digital video cameras (SONY HDR-CX470) from two angles: 1) the front, to capture the entire aquarium (central camera, 1 m away from the aquarium), and 2) the opposite side to the mirror, to capture the blind spots behind the side partition (side camera, 0.5 m away from the aquarium). The PVC tube and baited prawn were removed from the aquarium for the entire duration of the recording.

### Experimental procedure

The experiment was conducted in the laboratory at Osaka City University (Osaka Metropolitan University) between July 2020 and April 2021. We measured the total length of the focal fish by encasing them within a Ziplock bag and employing an exterior ruler to measure them to the nearest millimeter prior to their introduction to the experimental aquarium. All focal fish were habituated to the experimental aquaria for at least one week prior to the commencement of the experiment. To acclimate the fish to the photographs, two same-sized photographs were presented to them individually for a period of 3 min each, with a two-day interval between presentations. We did not include the data from this habituation phase in the results. After confirming that all fish exhibited aggressive behavior toward the photographs in the habituation phase, larger, same-sized, and smaller photographs were randomly presented to the fish individually for 3 min each, two days apart (first session). A previous study demonstrated that one week was sufficient for cleaner fish to establish MSR^22^; hence, the fish in the mirror treatment group were exposed to a mirror for one week. Following this mirror presentation, we conducted the mark test to assess whether the fish had established MSR. We followed the injection procedures outlined in previous studies^21,22^. In the mark test, all eight fish scraped their throats at least once. Two days after the test, additional larger, same-sized, and smaller photographs, distinct from those used in the first session, were presented to the fish that passed the mark test (second session). The procedure for presenting photographs was identical to that used in the first session. In the control treatment, photographs were presented in the same manner as in the mirror treatment. However, these seven fish were not exposed to the mirror between the two sessions of photograph presentation.

### Behavioral analysis

To evaluate the aggression levels of the focal fish toward the photographs, we examined their initial responses to the photographs shortly after their presentation. Although most individuals rarely attacked them physically (11 out of 15), they approached photographs (within 15 cm) and briefly gazed at them (ranging from 0.5 to 2.0 s) by rapidly and repeatedly turning their heads with their dorsal and caudal fins extended (Supplementary Movie 1). This series of spontaneous approaches toward a photograph suggested that the focal fish perceived the photograph as an opponent that could be excluded by attacking it. We considered the duration of this behavior as indicative of aggressiveness toward the photographs. A previous study demonstrated that fish rapidly discriminate a photograph (≤ 0.5 s) based on the face^41^; hence, we measured the initial duration of the focal fish orienting their head toward a photograph from the moment they first approached it until they left in both sessions using the annotation software ELAN ver. 5.9 (https://archive.mpi.nl/tla/elan/previous).

When a photograph was presented, the mirror-experienced fish occasionally ceased swimming or swam parallel along the mirror (5.25 times ± 0.98 SEM for larger photographs, 2.75 ± 0.73 for same-sized ones, and 1.00 ± 0.38 for smaller ones, Supplementary Movie 2). They often swam parallel to the mirror with their fins extended, similar to the lateral display^35^, a behavior performed to compare body size with that of an opponent^39^. Because cleaner fish that established MSR should be aware that their mirror image is a self-reflection, they should accurately recognize their body size based on their mirror image. We summed the durations of all parallel swimming in front of the mirror as an index to assess body size.

After leaving a position in front of the photograph, the fish that established MSR would voluntarily swim toward and observe the mirror before directly returning to the place in front of the photograph (Supplementary Movie 3). We assumed that the fish were comparing their body size with that of the photograph as the central partition prevented them from simultaneously observing both the mirror and the photograph (Fig. 2). We also quantified the frequency of swimming back and forth in front of the mirror and in the photographs.

All three behaviors discussed above were analyzed by a blinded observer (24 of 90 videos). The data analyzed by an experimenter and a blinded observer exhibited a high correlation (Pearson's correlation coefficient, initial interaction with the photograph: r = 0.79, n = 24; parallel swimming: r = 0.86, n = 12; reciprocal swimming: r = 0.89, n = 12).

### Statistics and reproducibility

Statistical analyses were conducted using R software version 4.3.0^64^. For the initial duration of interaction with the photographs, we used a linear mixed model (LMM) with treatments (mirror and control), sessions (first and second presentations with photographs), and photograph sizes (10% larger, the same size, and 10% smaller) as fixed factors and individual ID as a random factor^65^. Data were ln-transformed to satisfy the assumptions of normality. The residuals exhibited a normal distribution (Shapiro–Wilk normality test: W = 0.98, *p* = 0.14) and homoscedasticity of variances (Bartlett test: K^2^ = 10.12, df = 11, *p* = 0.52). The fixed effects accounted for 37% of the variance. Post-hoc analyses were conducted subsequent to any significant model results to identify the conditions influencing the overall significant outcomes by employing emmeans^66^. Effect sizes (partial η^2^) and their 95% confidence intervals (CI) were calculated for the LMMs and emmeans.

In addition to the initial interaction with the photograph, we collected data on the total duration of swimming parallel to the mirror and the frequency of swimming back and forth between the photograph and the mirror during the second session. Initially, we preferred to employ comprehensive models, including treatments and photograph sizes, as fixed factors. However, conducting this analysis was impossible because of the minimal variation in the data for the control group, in which more than 90% of the values were zero. Therefore, we initially summed the cumulative duration of parallel swimming or the frequency of reciprocal swimming for each individual over three trials with photographs of different sizes, followed by an exact Wilcoxon rank sum test^67^. Based on the significant outcomes of this test (parallel swimming: W = 0, *p* = 0.0003; reciprocal swimming: W = 2, *p* = 0.001), we analyzed only the data from the mirror treatment group to ascertain whether individuals exhibited differential responses to photographs of different sizes. We fitted only the photograph sizes in the LMM as a fixed factor with individual ID as a random factor. The data were subjected to ln (x + 0.001) transformation to meet the normality assumptions due to the presence of zero in the data. The parallel swimming model adhered to a normal distribution (W = 0.98, *p* = 0.93) and exhibited homoscedasticity (K^2^ = 1.4, df = 2, *p* = 0.50). Similarly, the reciprocal swimming model conformed to a normal distribution (W = 0.98, *p* = 0.95) and exhibited homoscedasticity (K^2^ = 1.8, df = 2, *p* = 0.40). Fixed effects explained 42% of the variance in parallel swimming and 39% in reciprocal swimming.

Analyses of the duration of parallel swimming and the frequency of reciprocal swimming highlighted the non-standard distribution of data in the control treatment. Therefore, we conducted an additional LMM analysis of the initial interaction duration with the photographs. This analysis was specifically limited to the second session of the mirror treatment, with photograph size and individual ID treated as fixed and random factors, respectively. The data underwent ln transformation to satisfy normality assumptions. The model conformed to a normal distribution (W = 0.93, *p* = 0.076) and demonstrated homoscedasticity (K^2^ = 0.14, df = 2, *p* = 0.93). Fixed effects explained only 14% of the variance, whereas the random effect (individual) exhibited a substantial influence by explaining 73.9% of the variance. These statistical outcomes may explain why the complete model showed only a two-way interaction between treatments and sessions rather than a three-way interaction involving treatments, sessions, and photograph sizes.

### Ethics statement

All of the experiments were conducted in accordance with the Animal Welfare Guidelines of the Japan Ethological Society and ARRIVE guidelines (https://arriveguidelines.org). The protocol for this study including the mark injection procedure received approval by the Animal Care and Use Committee of the Osaka Metropolitan University (No. S0088).

## Supplementary Information

Supplementary Information 1.

Supplementary Information 2.

Supplementary Video 1.

Supplementary Video 2.

Supplementary Video 3.

## Data Availability

All of the data are accessible within the main text and supplementary materials.
