# Supplementary material for: Cleaner fish with mirror self-recognition capacity precisely realize their body size based on their mental image
Source: Sci Rep. 2024 Sep 11;14:20202. doi: 10.1038/s41598-024-70138-7 (PMC11390716; doi:10.1038/s41598-024-70138-7)
Supplement: Supplementary file 1 — Supplementary Information 1. [file 41598_2024_70138_MOESM1_ESM.pdf]

Supporting Information

Cleaner fish with mirror self-recognition capacity precisely realize their body size based on their mental image

Taiga Kobayashi, Masanori Kohda, Satoshi Awata, Redouan Bshary & Shumpei Sogawa

Supplementary Results

To determine the optimal photograph size, we conducted a preliminary experiment in which eight mirror-naïve fish were presented with photographs scaled 10% and 20% larger than their actual sizes. The procedures for editing the photographs, setting up the tanks, and conducting the experimental procedure were consistent with those in the main experiment (refer to Stimulus photographs, Experimental aquaria, and Experimental procedure in the Materials and methods for further details). We measured the minimum distance at which the fish immediately approached the photograph upon presentation as a criterion for evaluating aggressiveness toward the photographs. The fish approached the 10% larger photograph significantly closer than the 20% larger one (exact Wilcoxon signed-rank test: $r = 0.87$, $V = 28$, $p = 0.016$). Based on this outcome, we used photographs with a 10% difference in size for the main experiment.

Supplementary Movie 1. An instance of aggressive behavior toward a photograph exhibited by a cleaner fish. This video demonstrates the aggressive response of a mirror-naïve focal fish shortly after a 10% larger photograph was presented. The photograph was attached to the front right wall of the aquarium from the outside (indicated by a photograph of a cleaner fish). This fish immediately approached the photograph and turned its head toward it with its fins spread. This behavior likely indicated that this fish did not perceive the photograph as a formidable threat. All mirror-naïve fish exhibited similar behaviors when exposed to photographs that were 10% larger, the same size, and 10% smaller. Fish confirmed to have attained mirror self-recognition (MSR) exhibited this behavior only when presented with a 10% smaller photograph.

Supplementary Movie 2. An instance of parallel swimming along a mirror exhibited by a cleaner fish. This video shows that a focal fish that established MSR either paused with its body oriented sideways toward the mirror or swam parallel to it when a 10% larger photograph was presented. The photograph was attached to the front right wall of the aquarium from the outside (indicated by a photograph of a fish), and a mirror was positioned diagonally opposite the photograph (depicted by a white dashed line). These behaviors resemble lateral displays and may indicate that this fish assesses body size based on its mirror image. All of the mirror-experienced fish repeatedly displayed these behaviors, particularly when a larger photograph was presented.

Supplementary Movie 3. An instance of swimming back and forth between a photograph and a mirror exhibited by a cleaner fish. This video demonstrates that a focal fish, which established MSR, swam back and forth seven times between a mirror and a photograph 10% larger. The photograph was positioned on the front right exterior wall of the aquarium (indicated by a photograph of a fish), and a mirror was placed diagonally opposite the photograph (depicted by a white dashed line). This behavior suggests that this fish engages in size comparison between its own body and an enlarged photograph through reciprocal swimming. All mirror-experienced fish repeatedly exhibited this behavior, particularly in the presence of a larger photograph.

Supplementary Data.

Dataset 1: Dataset for Fig. 3. Initial duration of aggressive interactions exhibited by the focal fish toward the photographs.

Dataset 2: Dataset for Fig. 4a. Cumulative duration of parallel swimming along the mirror.

Dataset 3: Dataset for Fig. 4b. Frequency of swimming back and forth between the photograph and the mirror.

Dataset 4: Total length of all focal fish and the size of photographs.

Dataset 5: The frequency of throat scraping in the mark test.

Dataset 6: Dataset for blind analysis. T.K. is the experimenter, and N.K. is the blind observer.

Dataset 7: Dataset for preliminary trials.
